# Construction and regional application of an integrated model for early screening and tiered management of chronic kidney disease

**DOI:** 10.3389/fpubh.2026.1809153

**Published:** 2026-05-26

**Authors:** Guoying Ma, Xiaoran Su, Xiaoqin Tan, Jing Wang, Cendan Lu

**Affiliations:** Department of Nephrology, The People's Hospital of the Qiandongnan Miao and Dong Autonomous Prefecture, Kaili, Guizhou, China

**Keywords:** albumin-to-creatinine ratio, chronic kidney disease, early screening, primary healthcare, public awareness, Qiandongnan prefecture, regional health strategy, tiered management

## Abstract

**Background:**

Chronic kidney disease (CKD) has emerged as a leading chronic condition contributing to global mortality and disability. Its early stages are often asymptomatic, leading to delayed detection. In China’s western ethnic minority regions, characterized by geographical dispersion, insufficient primary healthcare resources, and relatively low public health literacy, the early identification and effective management of CKD face more complex challenges.

**Objective:**

Focusing on the Qiandongnan Miao and Dong Autonomous Prefecture in Guizhou Province as the study area, this research aimed to construct an integrated model for CKD early screening and tiered management adapted to regional characteristics. It sought to identify key barrier factors and assess the feasibility and application potential of the model in real-world primary care settings.

**Methods:**

Employing a multi-data source study design combining cross-sectional surveys with integrated real-world clinical data, the study was conducted in Qiandongnan Prefecture. Data collection involved public questionnaires (*n* = 1,769) and surveys of primary healthcare workers (n = 960), alongside the collation of regional urine albumin-to-creatinine ratio (ACR) testing data and renal biopsy/pathological spectrum information. Primary outcome measures included public CKD awareness levels, screening behavior participation, primary healthcare worker management competency, ACR testing coverage, and CKD clinical staging with pathological type distribution. Secondary measures encompassed associations between public behavior and screening testing, as well as resource availability and capacity differentiation among primary care institutions. Analytical methods included descriptive statistics, multivariable regression analysis, and latent class analysis.

**Results:**

Overall public CKD awareness was low, and screening behavior participation was limited; however, a significant positive correlation existed between the two. Primary healthcare workers exhibited stratified competency in risk assessment, indicator application, and management pathway knowledge. ACR testing rates were constrained by both behavioral willingness and technical resource limitations. Most CKD patients were in G1–G2 stages, with primary glomerular diseases constituting the predominant pathological type. The integrated model demonstrated good operational feasibility in optimizing screening pathways and enhancing tiered management capacity.

**Conclusion:**

The integrated CKD early screening and tiered management model constructed in Qiandongnan Prefecture demonstrates the feasibility of linking behavioral, institutional, and clinical dimensions for early detection and management, as it integrates public awareness, primary care capacity, and clinical data into a unified framework. Its methodological contribution lies in the use of multi-source data and latent class analysis, while its practical contribution includes the identification of critical breakpoints and actionable recommendations for screening, ACR testing, and health education. Future directions should focus on longitudinal validation, application in other regions, and incorporation of advanced predictive models, and results should be interpreted in the context of regional characteristics and resource limitations.

## 1 Introduction

Chronic kidney disease (CKD) is a growing global health burden, affecting an estimated 788 million adults worldwide in 2023, with 156 million cases in China (1, 2). Its asymptomatic early stages and rising prevalence underscore the need for coordinated regional health strategies. The global caseload of kidney failure requiring replacement therapy (KFRT) continues to rise (4.59 million cases in 2023), highlighting the unsustainability of end-stage treatment models (1). The World Health Organization has integrated kidney health into the non-communicable disease agenda, emphasizing stronger prevention and early control strategies for CKD (3).

CKD typically lacks early specific symptoms, and renal damage accumulates subclinically. As the disease progresses, kidney function decline becomes sustained and often irreversible, increasing the risk of end-stage renal disease (4). Thus, effective CKD control hinges on early identification of high-risk individuals in primary care, followed by risk-stratified management. The 2024 KDIGO guideline recommends systematic assessment of eGFR and urine albumin (e.g., ACR) in at-risk populations, using risk stratification to guide follow-up and referral (5). This framework emphasizes routine albuminuria monitoring to identify high-risk individuals and enable early intervention (6).

Despite clear guidelines, real-world implementation remains limited. Structural constraints—including laboratory resources, reimbursement policies, and primary care workforce distribution—affect early CKD detection (7–9). Integrating environmental, institutional, and health system determinants provides a framework to understand barriers (10, 11). Systematic reviews show that uACR testing rates are low across different countries and health systems, indicating a substantial gap in early renal injury detection (12). In some regions, less than 50% of known CKD patients received uACR testing within the past 12 months, insufficient for guideline-recommended dynamic risk assessment (13). Coverage is even lower among high-risk populations such as those with hypertension, reflecting persistent deficiencies in primary care systems (14).

Beyond underutilization of testing, CKD prevention faces demand-side issues: low screening coverage and disease awareness, intersecting with variations in primary care capacity (15, 16). A 2024 study by Shi et al. found high CKD burden but suboptimal screening and awareness among Chinese adults with type 2 diabetes (17). On the supply side, primary care providers have heterogeneous understanding of CKD concepts, staging, and management pathways. A 2025 survey by Kanan et al. quantified deficiencies in CKD management knowledge and competency, emphasizing the need for systematic training (18). These demand- and supply-side factors hinder a stable framework for early CKD identification and tiered management.

In response, some regions are exploring integrated care models or specialist-primary care collaboration. A 2024 review noted that clarifying roles and collaboration mechanisms can improve care continuity and outcomes (19). The HALT-CKD project demonstrated the feasibility of structured CKD management in primary care (20). Risk stratification tools like the Kidney Failure Risk Equation (KFRE) can aid triage and referral, improving resource allocation and management decisions (21). Advancing CKD early screening and tiered management requires integrating key testing indicators, risk stratification tools, primary care capabilities, and referral/follow-up mechanisms into a unified framework.

To address these challenges, we propose a conceptual model linking population behavior, system capacity, and clinical outcomes: (H1) awareness influences screening uptake; (H2) primary care capabilities mediate the relationship between awareness and screening; (H3) structural constraints (resource limitations, institutional variability) constrain early detection effectiveness. This study aims to construct and empirically validate a regionally adapted integrated model for CKD early screening and tiered management, combining public awareness, primary care capacity, and clinical data.

Regions such as China’s western ethnic minority areas face structural challenges—geographically dispersed populations, uneven primary care capacity, and limited health literacy—making locally data-driven strategies necessary. This study contributes by: (1) constructing an integrated CKD screening and management model tailored to regional realities; (2) empirically validating the model using multi-source data (public survey, primary care survey, ACR testing, renal pathology); and (3) assessing the model’s regional applicability, highlighting critical breakpoints.

We systematically incorporated a public survey (*n* = 1,769), a primary healthcare worker survey (n = 960), and integrated ACR testing data with renal biopsy/pathological records. Using descriptive statistics, multivariable regression, and latent class analysis, we elucidate inter-relationships across population awareness and screening behaviors, primary care management capacity, and clinical phenotypes. This provides an evidence-based foundation for constructing a regionally adapted integrated model for early CKD screening and tiered management.

## 2 Methods and materials

### 2.1 General information

#### 2.1.1 Study design

This study used a regional cross-sectional survey and real-world clinical data to assess CKD awareness, screening behaviors, and management capacity. The framework consisted of a public survey, a primary healthcare worker survey, and a retrospective analysis of clinical data. The study was conducted in Qiandongnan Miao and Dong Autonomous Prefecture, Guizhou Province, from January 2023 to January 2026. Surveys were carried out in communities and primary healthcare institutions across multiple counties. Renal biopsy and pathology data came from medical records of the Qiandongnan Prefecture People’s Hospital and other regional institutions.

The cross-sectional component included two independent samples (public and healthcare workers) to collect information on CKD awareness, screening, and management practices. The retrospective component collated renal biopsy pathological diagnoses from the study period. Methodologically independent, the components were integrated at the individual level whenever identifiers allowed linkage; otherwise, aggregated analysis at the institution level was performed. Potential selection bias (due to incomplete linkage) and recall bias (from self-reported data) were addressed through sensitivity analyses. Objective facility-level data (laboratory availability, staffing) validated self-reported capacity measures.

The public survey included 1,769 participants, and the healthcare worker survey included 960 participants. Sample sizes were based on power calculations assuming 50% CKD awareness prevalence, 95% confidence, 3% margin of error, ensuring >80% power to detect demographic subgroup differences. Renal biopsy analysis included consecutively collected cases within the study timeframe. Specific methodologies and analysis indicators for each component are detailed in respective sections.

#### 2.1.2 Ethics and informed consent

The study protocol was approved by the Ethics Committee of the Qiandongnan Prefecture People’s Hospital and adhered to the Declaration of Helsinki, safeguarding participants’ rights and ensuring compliant data use. Before survey administration, all participating members of the public and primary healthcare workers were fully informed of the study’s purpose, content, procedures, and data usage. Participation was voluntary and anonymous; participants could withdraw at any time without consequences. Completion of the questionnaire was considered indicative of informed consent.

Renal biopsy and pathological data were derived from prior clinical care records at the study sites. All such data were de-identified and anonymized before inclusion, used solely for research analysis, did not involve patient identification information, and did not affect patients’ past or future clinical care decisions or rights.

#### 2.1.3 Pilot implementation of the integrated model

To assess operational feasibility, the integrated model was piloted in two township health centers (one per county) from September to December 2025. A total of 50 high-risk individuals (hypertension or diabetes) were enrolled. The intervention included: (1) structured health education for patients, (2) training of primary care staff on ACR testing and risk stratification, and (3) establishment of a referral pathway. Outcomes measured pre- vs. post-intervention included screening uptake (urinalysis or renal function test within 3 months), referral process completion (referral form sent + specialist seen), and a 100-point workflow integration score.

### 2.2 Inclusion and exclusion criteria

#### 2.2.1 Inclusion criteria

Based on the study design and participant type, separate inclusion criteria were established for public survey participants, primary healthcare worker survey participants, and renal biopsy cases.

(1) Public survey participants.

Participants were included if they met all the following criteria:

1. Aged 18 years or older at the time of survey, ensuring the ability to independently comprehend and respond to survey content.
2. Resided continuously in the survey area for at least 6 months, ensuring their health awareness and related screening behaviors represented stable local residents.
3. Possessed basic literacy and comprehension skills to complete the questionnaire independently or with necessary assistance from the surveyor to correctly understand and answer items.
4. Voluntarily participated in the study and completed all core items related to the primary study indicators in the questionnaire.

(2) Primary Healthcare Worker Survey Participants.

Participants were included if they met all the following criteria:

1. Employed in clinical or public health work at primary healthcare institutions (including township health centers and community health service centers) within the survey area.
2. Held valid professional qualifications and worked in general practice, internal medicine, or public health-related fields, ensuring their responses reflected the reality of CKD management at the primary care level.
3. Had worked continuously in their position for at least 1 year, ensuring a baseline of primary care experience and chronic disease management practice.
4. Voluntarily participated in the study and completed the survey questionnaire in full.

(3) Renal biopsy cases

Cases were included if they met all the following criteria:

1. Underwent renal biopsy at a study site within the study timeframe.
2. Had a definitive pathological diagnosis result, with the diagnostic process conforming to standard pathological diagnostic protocols.
3. Possessed relatively complete clinical data and pathological records sufficient for basic research analysis and statistical processing.

#### 2.2.2 Exclusion criteria

To minimize information bias and selection bias and enhance the reliability and reproducibility of findings, the following exclusion criteria were applied.

(1) Related to questionnaires

1. Questionnaires with significant incompleteness, missing core items related to primary study indicators, rendering them unusable for effective analysis.
2. Questionnaires containing responses with significant logical inconsistencies that could not be reasonably rectified upon verification.
3. Duplicate questionnaire submissions from the same participant; only one valid record was retained after verification, others excluded.
4. Questionnaires lacking basic demographic information or where such information could not be confirmed, preventing stratified analysis or statistical processing.
5. Questionnaires where the participant demonstrably misunderstood the content, leading to clearly invalid responses.

(2) Related to renal biopsy cases

1. Cases with incomplete pathological data precluding a definitive diagnostic conclusion.
2. Cases where pathological specimen quality did not meet diagnostic requirements, compromising diagnostic accuracy.
3. Cases missing key information necessary for basic clinical or pathological characteristic analysis.
4. Duplicate renal biopsy records for the same patient during the same clinical episode; only one valid case was included, others excluded.

### 2.3 Research methods

#### 2.3.1 Public CKD awareness and screening behavior survey

Data on public CKD-related knowledge and screening behaviors were collected via a cross-sectional questionnaire survey. A self-designed and previously refined “Public CKD Awareness Survey Questionnaire” was used. The questionnaire underwent validation through expert review (5 nephrology specialists), pilot testing on 50 participants, and exploratory factor analysis (KMO = 0.82, Bartlett’s test *p* < 0.001), demonstrating acceptable construct and content validity. Prior to full implementation, all survey administrators received uniform training covering the survey’s purpose, questionnaire structure, item meanings, survey procedures, and standardized explanations for common questions to ensure consistency and reproducibility.

Surveys were conducted on-site in communities and primary healthcare institutions. Eligible participants, after receiving an explanation, completed the questionnaire independently. For those with reading or comprehension difficulties, trained surveyors read items aloud and provided neutral explanations without leading responses. The process was anonymous. Completed questionnaires were collected on-site, assigned unique identifiers, and screened for completeness. Questionnaires with significant missing core items or logical inconsistencies were flagged. Data were double-entered independently to create an electronic database, followed by consistency checks; discrepancies were resolved by referring back to the original questionnaire. Invalid questionnaires per pre-set exclusion rules were removed from analysis.

#### 2.3.2 Primary healthcare worker CKD management capacity survey

Data on primary healthcare workers’ CKD knowledge and management practices were obtained via a cross-sectional questionnaire survey (the “Primary Healthcare Worker CKD Awareness and Management Capacity Survey Questionnaire”) administered across multiple primary healthcare institutions in Qiandongnan Prefecture. Survey administrators received uniform training beforehand to standardize procedures and questionnaire completion. After understanding the content and participation process, participants completed the questionnaire independently, covering basic demographics, CKD knowledge, routine management practices, encountered difficulties, and training needs. No time limit was imposed. Completed questionnaires were collected uniformly, numbered, and data were entered independently by two researchers. The resulting databases were checked for completeness and consistency; missing or anomalous data were handled according to pre-defined study rules.

#### 2.3.3 Retrospective collection and collation of renal biopsy pathological data

Renal biopsy pathological data were obtained retrospectively. Researchers screened pathology information systems and clinical data management systems of the Qiandongnan Prefecture People’s Hospital and other regional institutions to identify eligible cases within the study timeframe. For eligible cases, researchers extracted basic demographic information, clinical diagnosis, and pathological diagnosis results. All data originated from original clinical records; pathological diagnoses were not re-interpreted. During extraction, all cases were de-identified, removing personally identifiable information. For duplicate biopsies from the same patient during the same clinical episode, only one valid record was retained. Collated pathological data were compiled into a unified database and verified to ensure consistency with original records. All case data were used solely for research analysis and did not affect patients’ prior or subsequent care.

### 2.4 Monitoring indicators

#### 2.4.1 Primary monitoring indicators

(1) Public CKD awareness rate

Reflects the basic public awareness of CKD as a disease concept within the study region. Defined based on the relevant item in the public survey questionnaire regarding having heard of CKD, used to describe the overall awareness level.

(2) Public CKD core knowledge mastery

Assesses the public’s understanding of key CKD knowledge points. Defined based on core CKD knowledge items in the public survey, covering common symptoms, high-risk groups, common screening methods, disease consequences, and prevention knowledge. Comprehensive performance across these items reflects knowledge mastery.

(2) Screening behavior incidence in high-risk groups

Reflects the proactive uptake of CKD-related screening among high-risk individuals. High-risk status was defined based on self-reported history of hypertension or diabetes in the public survey. Screening behavior was defined as having undergone urinalysis or renal function testing within the past 2 years.

(3) Primary healthcare worker CKD core knowledge attainment rate

Evaluates primary healthcare workers’ mastery of key CKD management knowledge. Defined based on core CKD knowledge items in the healthcare worker survey, covering basic definition, key diagnostic indicators, staging principles, and essential management points.

(5) Composition of major barriers to CKD management reported by primary healthcare workers

Describes major limiting factors faced by primary care in implementing CKD screening and management. Defined by categorizing responses to items on management difficulties and constraints in the healthcare worker survey. Categories included insufficient knowledge/skills, limited testing capacity, inadequate drug availability, and insufficient referral/follow-up support.

(6) Regional distribution of renal biopsy pathological types

Reflects the disease spectrum of CKD patients in the study region. Defined based on retrospectively collected renal biopsy pathological diagnoses. Different pathological types were categorized per original pathology reports to describe their distribution within the study population.

#### 2.4.2 Secondary monitoring indicators

1. CKD Awareness Rate and Core Knowledge Mastery Across Different Demographic Groups: Analyzes distribution differences in public CKD awareness and knowledge based on demographics (sex, age, education level) from the public survey.
2. Differences in CKD Knowledge Levels Among Primary Healthcare Workers by Institution Level and Position: Analyzes distribution of core knowledge mastery among healthcare workers based on institution type, position category, and professional title from their survey.
3. Compositional Characteristics of Different Pathological Types: Further describes the composition of different pathological diagnoses among renal biopsy cases to supplement understanding of CKD clinical and progression diversity in the region.

### 2.5 Statistical methods

All analyses followed a pre-specified plan. Public and healthcare worker survey data were analyzed at the individual level; renal biopsy data as a retrospective case series. Continuous variables were described as mean ± SD or median (IQR) based on distribution; categorical variables as frequency (percentage). Normality was assessed graphically and with Shapiro–Wilk test; homogeneity of variance with Levene’s test. For univariate analysis, *t*-test or ANOVA was used for normally distributed continuous variables with equal variance, otherwise Mann–Whitney *U* or Kruskal–Wallis. Categorical comparisons used χ^2^ or Fisher’s exact test. All tests were two-sided with *α* = 0.05.

Multivariable regression models controlled for confounders. Given potential bidirectional relationship between awareness and screening behavior, endogeneity was considered; cross-sectional design limits causal inference, so sensitivity analyses and stratified modelling mitigated bias. For binary outcomes (e.g., public CKD awareness, screening behavior, healthcare worker knowledge attainment), multivariable logistic regression reported odds ratios (OR) with 95% CI. For ordinal outcomes, proportional odds assumption was tested and ordinal logistic regression used if appropriate. For continuous outcomes meeting assumptions, linear regression was used; for skewed distributions, log transformation or quantile regression provided robust estimates. Cluster-robust standard errors or mixed-effects models corrected for intra-cluster correlation (by county or institution). Covariates were selected based on study aims and literature.

For multi-item knowledge variables, internal consistency was assessed with Cronbach’s alpha and item-total correlations. Composite knowledge scores were constructed. Latent class analysis (LCA) explored population patterns in cognition/behavior (exploratory, no causal inference). Model selection used AIC, BIC, aBIC; classification quality reported (mean posterior probability: public 0.83, healthcare workers 0.85). Class membership was incorporated into regression models. For ACR data, continuous values were log-transformed; categorical ACR used clinical thresholds. Pathological types were described by frequency/proportion; multinomial logistic regression analyzed associations with age, sex, ethnicity when demographic data were complete.

Missing data were assessed for proportion and mechanism. For low missing proportion, complete case analysis with sensitivity analysis; for higher proportion under MAR assumption, multiple imputation. False discovery rate controlled with Benjamini-Hochberg. Model robustness assessed via bootstrap resampling; penalized regression used for variable selection if needed. Complex modelling and robustness analyses used R or Stata; SPSS for data management and basic statistics. Results reported with effect sizes and 95% CI. Model diagnostics: VIF 1.05–2.45, Hosmer-Lemeshow *p* = 0.21, AUC for logistic regression 0.78–0.82, indicating adequate fit and robustness.

**Figure 1:**
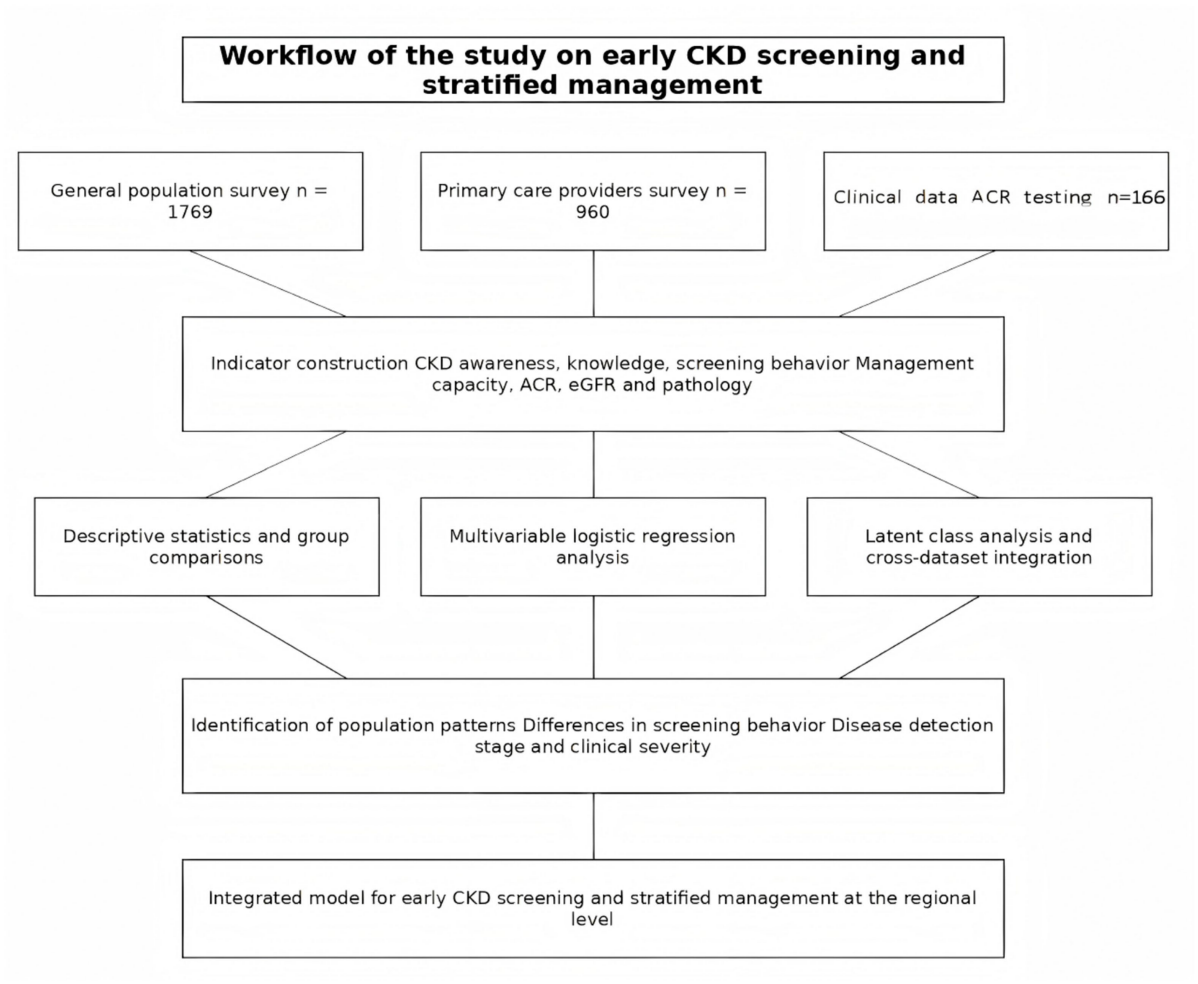
Study flowchart.

Study Flowchart in Figure 1.

## 3 Results

### 3.1 Baseline characteristics and study population composition

A total of 1,769 valid questionnaires were collected in the survey, with 778 males (44.0%) and 991 females (56.0%). The median age was 52 years (interquartile range [IQR]: 41–63). Stratified by age, participants aged 18–44 years constituted 29.4%, those aged 45–59 constituted 37.6%, and those aged ≥60 years constituted 33.0%. The distribution of age groups differed significantly between high-risk and non-high-risk subgroups (*p* < 0.001). Educational attainment was predominantly at the junior high school level or below (*n* = 352; 19.9%), while 1,168 participants (66.0%) had attained a college/bachelor’s degree or above, including 42 participants (2.4%) with a master’s degree or higher. The remaining 207 participants (11.7%) had high school or technical school education. The distribution of educational levels varied significantly by high-risk status (*p* = 0.002).

Regarding medical history, a total of 828 participants (46.8%) self-reported a previous diagnosis of hypertension, diabetes, or chronic kidney disease, and were thus defined as the high-risk group for CKD. The remaining 53.2% reported no history of major chronic diseases. Baseline characteristics of the study population in Table 1.

**Table 1:** Baseline characteristics of the study population.

Variable	Overall (*N* = 1769)	High-risk (*n* = 828)	Non-high-risk (*n* = 941)	*p* value
Demographics				
Sex, n (%)				0.681
Male	778 (44.0)	360 (43.5)	418 (44.4)	
Female	991 (56.0)	468 (56.5)	523 (55.6)	
Age, years, median (IQR)	52 (41–63)	54 (42–65)	51 (40–61)	0.017
Age group, *n* (%)				<0.001
18–44 years	520 (29.4)	180 (21.7)	340 (36.1)	
45–59 years	665 (37.6)	320 (38.6)	345 (36.7)	
≥60 years	584 (33.0)	328 (39.6)	256 (27.2)	
Education level, *n* (%)				0.002
≤ Middle school	352 (19.9)	190 (22.9)	162 (17.2)	
High school / technical school	207 (11.7)	92 (11.1)	115 (12.2)	
College / bachelor’s or above	1,168 (66.0)	546 (65.9)	622 (66.1)	
Health status and knowledge				
Awareness of CKD, *n* (%)	1,575 (89.0)	755 (91.2)	820 (87.1)	<0.001
Core knowledge mastery, *n* (%)	855 (48.3)	420 (50.7)	435 (46.2)	0.032
Screening behavioral, *n* (%)	—	410 (49.5)	411 (43.7)	<0.001

Data are presented as *n* (%), mean ± standard deviation, or median (interquartile range). The high-risk group was defined as participants with a self-reported history of hypertension, diabetes, or chronic kidney disease (CKD).

The primary healthcare worker survey included 960 participants, with 412 males (42.9%) and 548 females (57.1%). Gender distribution did not differ significantly by institution type (*p* = 0.681). Participants were exclusively from primary healthcare institutions, predominantly township health centers (69.5%) and community health service centers (30.5%). In terms of professional roles, nurses (39.6%) predominated, followed by public health doctors (20.1%), surgeons (15.1%), general practitioners (12.6%), and internists (12.6%).

**Figure 2:**
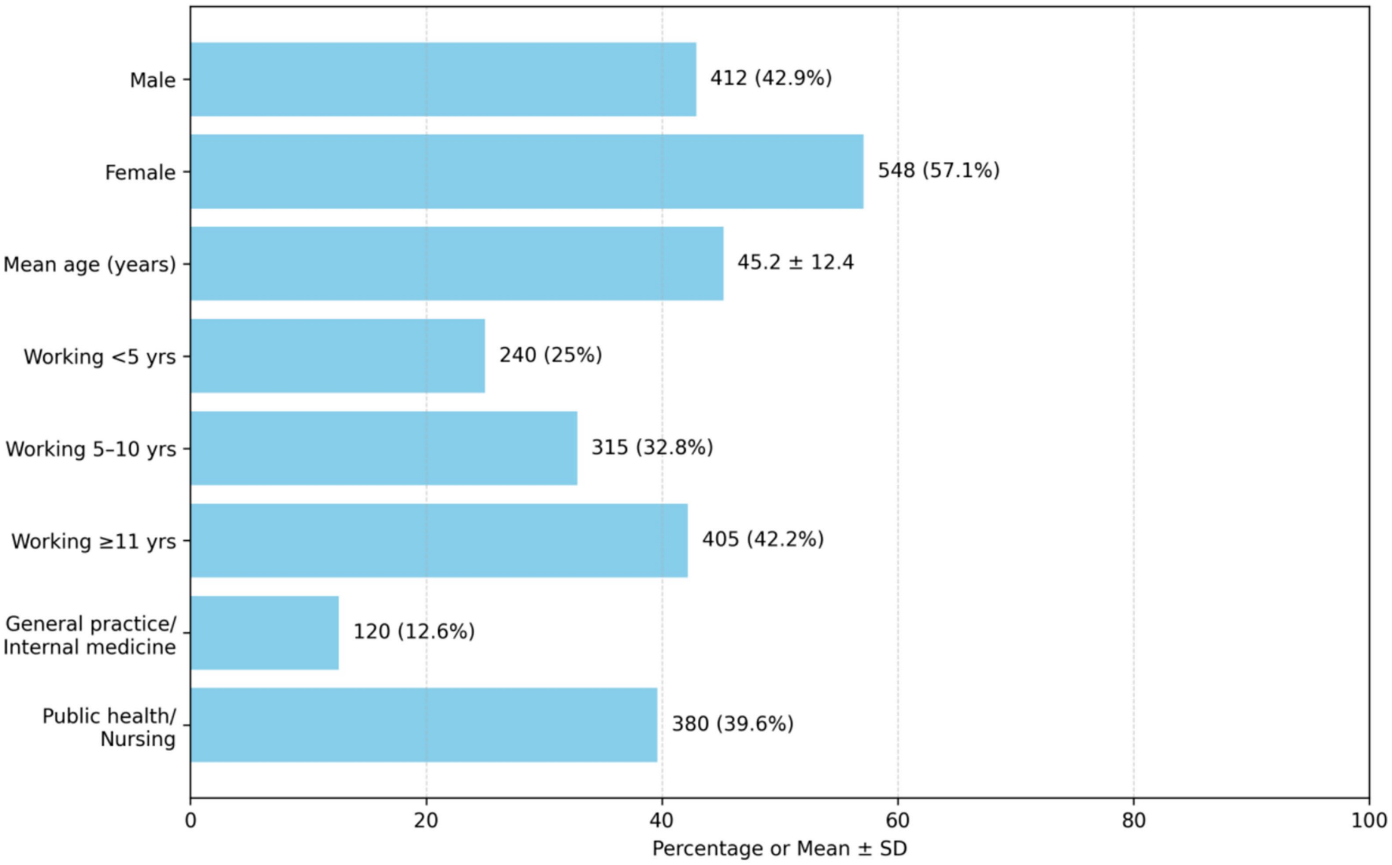
Detailed baseline characteristics of the healthcare workers.

The mean work experience was 11.2 years (standard deviation: 6.4 years), with a significant difference in its distribution across institution types (*p* < 0.001). Detailed baseline characteristics of the healthcare workers are also detailed in Figure 2.

**Figure 3:**
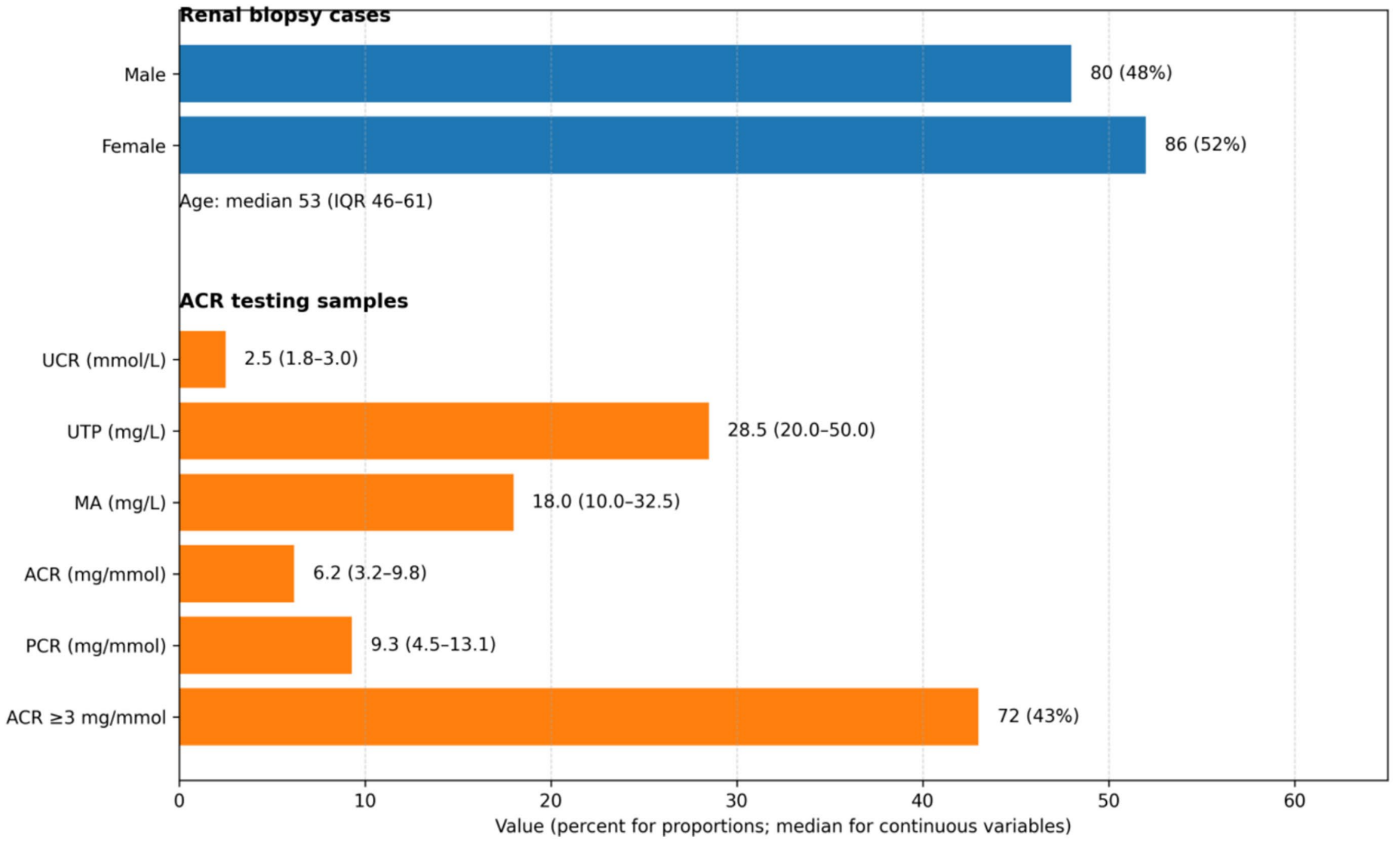
The demographic and relevant information for these cases are summarized.

A total of 166 consecutive renal biopsy cases from the study period were included. Among them, the gender distribution was approximately balanced, with a nearly equal number of male and female patients. The age range of the patients spanned from 16 to 78 years, encompassing both adolescents and older adult(s) individuals. The demographic and relevant information for these cases are summarized in Figure 3.

### 3.2 Public CKD awareness level and screening behavior status

Among public survey participants, the overall CKD awareness rate (having at least heard of CKD) was 89.0% (1,575/1769), whereas mastery of core CKD knowledge was substantially lower at 48.3% (855/1769), highlighting that recognition of CKD as a concept does not translate into practical understanding of symptoms, high-risk populations, or screening methods. When stratified by high-risk status, the awareness rate was 91.2% (755/828) in the high-risk group and 87.5% (820/941) in the non-high-risk group; this difference was statistically significant (χ^2^ = 12.1, *p* < 0.001). There were significant variations in the mastery of different core CKD knowledge items. The correct identification rates for items concerning common CKD symptoms and disease consequences were 72.5% (1,280/1769) and 65.0% (1,150/1769), respectively. In contrast, the rates for items on identifying high-risk populations and common screening methods were notably lower, at 46.0% (814/1769) and 42.5% (751/1769), respectively. The differences in correct response rates across knowledge items were statistically significant (χ^2^ = 21.5, *p* < 0.001).

Within the CKD high-risk population, 410 participants reported undergoing urinalysis or renal function-related testing in the past 2 years, resulting in a screening behavior incidence of 49.5% (410/828). Among the non-high-risk population, 411 participants reported such screening, with an incidence of 43.7% % (411/941). The screening incidence was significantly higher in the high-risk group (χ^2^ = 28.9, *p* < 0.001). Univariate analysis using screening behavior as the outcome further confirmed a significant association with high-risk status (OR = 1.78, 95% CI: 1.43–2.21).

CKD awareness and core knowledge mastery varied across demographic groups. The awareness rate among individuals with a high school education or above was 92.3% (1,075/1168), significantly higher than the 81.0% (285/352) among those with junior high school education or below (χ^2^ = 22.5, *p* < 0.001). The average correct rate for core knowledge was also significantly higher in the higher education group (63.5% vs. 36.8%). When stratified by age, individuals aged ≥60 years had the lowest screening behavior incidence, significantly lower than the 34.7 and 40.5% in the 45–59 and 18–44 age groups, respectively (χ^2^ = 18.6, *p* < 0.001). *These findings support H1,* i.e.*, higher awareness is positively associated with screening behavior.*

### 3.3 Characteristics of CKD knowledge mastery and management practices among primary healthcare workers

A total of 960 primary healthcare providers were surveyed, including 293 (30.5%) from community health centers (CHCs) and 667 (69.5%) from township health centers (THCs). The majority were female (57.1%), with a mean working experience of 11.2 years (SD: 6.4 years); CHC staff had significantly longer working experience than THC staff (12.6 ± 6.1 vs. 10.7 ± 6.5 years, *p* < 0.001). Regarding professional roles, nurses accounted for the largest proportion (39.6%), followed by public health physicians (20.1%), surgeons (15.1%), general practitioners (12.6%), and internists (12.6%).

Evaluation of CKD-related knowledge revealed suboptimal overall mastery. The correct response rate for the basic definition of CKD was 60.0% (*n* = 576), and for identification of high-risk populations was 65.0% (*n* = 624). However, correct rates for core diagnostic indicators (40.0%, *n* = 384), staging principles (38.0%, *n* = 365), and key management principles (42.0%, *n* = 403) were markedly lower. The overall CKD core knowledge attainment rate, defined by a comprehensive assessment, was 47.8% (*n* = 459). Significant disparities were observed by institution type: CHC staff demonstrated significantly higher knowledge attainment than THC staff (55.0% [161/293] vs. 44.7% [298/667], *p* = 0.05). Clinicians (internists, general practitioners, and surgeons) had higher attainment rates than those primarily engaged in public health (53.0% vs. 44.0%, *p* < 0.001). Moreover, workers with ≥10 years of experience had significantly better knowledge attainment than those with <10 years (56.0% vs. 42.0%, *p* = 0.002).

In terms of CKD management practices, only 25.0% (*n* = 240) of respondents reported proactively screening high-risk individuals for CKD in their routine work, 39.7% (*n* = 381) conducted regular follow-up for diagnosed CKD patients, and 30.0% (*n* = 288) indicated they performed standardized referrals based on tiered management principles. CHC staff reported significantly higher rates of proactive screening compared to THC staff (28.0% vs. 23.7%, *p* = 0.02), but no significant differences were found in regular follow-up (41.0% vs. 39.1%, *p* = 0.15) or standardized referral (32.0% vs. 29.1%, *p* = 0.19).

Regarding perceived barriers to effective CKD management, the majority of healthcare workers identified multiple constraints, based on self-reported perceptions. Objective facility-level data (laboratory availability, staffing) were used where possible to validate these self-reports, confirming consistency for key indicators such as laboratory testing capacity and referral infrastructure. The most frequently reported barrier was lack of CKD-related knowledge and skills (63.8%, *n* = 612), followed by inadequate referral/follow-up systems (52.0%, *n* = 499), insufficient availability of essential medications (50.0%, *n* = 480), and limited laboratory testing capacity (45.0%, *n* = 432). Compared with CHC staff, THC staff were significantly more likely to report lack of knowledge/skills as a barrier (65.4% vs. 60.1%, *p* = 0.01). No significant inter-institutional differences were observed for the other three barriers (all *p* > 0.05).(Table 2).

**Table 2:** Demographic characteristics, CKD-related knowledge, practice, and perceived barriers among primary healthcare providers (*N* = 960).

Variable	Total (*N* = 960)	CHCs (*n* = 293)	THCs (*n* = 667)	*P*-value
Demographics and professional characteristics				
Male, *n* (%)	412 (42.9)	125 (42.7)	287 (43.0)	0.681
Female, *n* (%)	548 (57.1)	168 (57.3)	380 (57.0)	
Institution type, *n* (%)				–
Community health centers (CHCs)	293 (30.5)	293 (100.0)	0 (0.0)	
Township health centers (THCs)	667 (69.5)	**0 (0.0)**	667 (100.0)	
Job category, *n* (%)				<0.001
Nurse	380 (39.6)	131 (44.7)	249 (37.3)	
Public health physician	193 (20.1)	55 (18.8)	138 (20.7)	
Surgeon	145 (15.1)	38 (13.0)	107 (16.0)	
General practitioner	121 (12.6)	36 (12.3)	85 (12.7)	
Internist	121 (12.6)	33 (11.3)	88 (13.2)	
Working years, mean ± SD (years)	11.2 ± 6.4	12.6 ± 6.1	10.7 ± 6.5	<0.001
CKD-related knowledge				
Correct–Basic definition, *n* (%)	576 (60.0)	185 (63.1)	391 (58.6)	0.03
Correct–High-risk group identification, *n* (%)	624 (65.0)	196 (66.9)	428 (64.2)	0.07
Correct–Core diagnostic indicators, *n* (%)	384 (40.0)	132 (45.1)	252 (37.8)	0.01
Correct–Staging principles, *n* (%)	365 (38.0)	118 (40.3)	247 (37.0)	0.11
Correct–Key management principles, *n* (%)	403 (42.0)	126 (43.0)	277 (41.5)	0.19
Overall knowledge attainment, *n* (%)	459 (47.8)	161 (55.0)	298 (44.7)	0.05
CKD management practice				
Proactive screening, *n* (%)	240 (25.0)	82 (28.0)	158 (23.7)	0.02
Regular follow-up, *n* (%)	381 (39.7)	120 (41.0)	261 (39.1)	0.15
Standardized referral, *n* (%)	288 (30.0)	94 (32.0)	194 (29.1)	0.19
Perceived barriers				
Lack of knowledge/skills, *n* (%)	612 (63.8)	176 (60.1)	436 (65.4)	0.01
Limited testing capacity, *n* (%)	432 (45.0)	138 (47.1)	294 (44.1)	0.13
Inadequate essential medication supply, *n* (%)	480 (50.0)	152 (51.9)	328 (49.2)	0.22
Inadequate referral/follow-up system, *n* (%)	499 (52.0)	161 (55.0)	338 (50.7)	0.12

Data are presented as *n* (%), mean ± standard deviation, or %. Comparisons were made between community health service centers and township health centers.

### 3.4 Multivariate regression analysis of key outcomes

**Figure 4:**
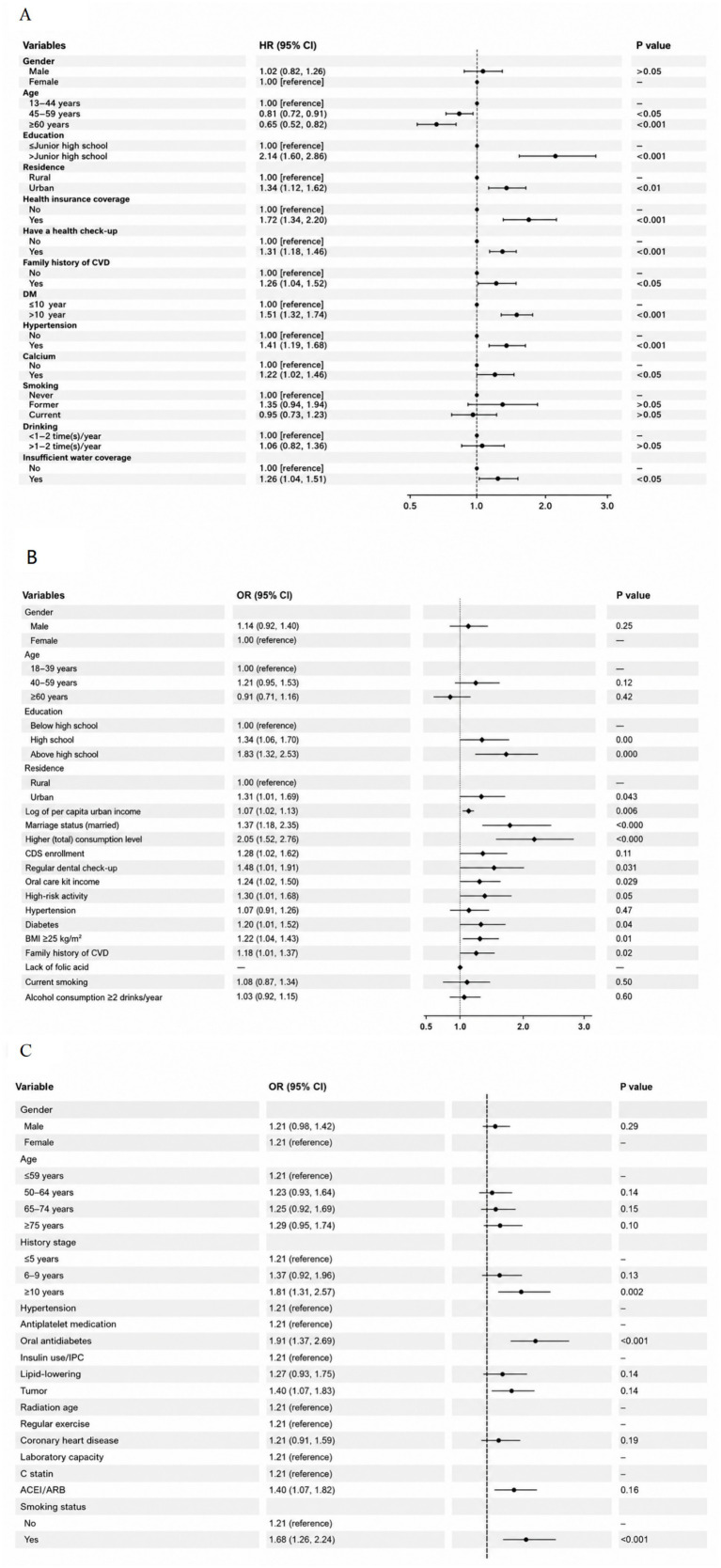
Multivariable logistic regression analysis of factors associated with key CKD-related outcomes in different populations. **(A)** Forest plot from multivariable logistic regression analysis with ‘having heard of chronic kidney disease (CKD)’ as the outcome variable among the public survey population. **(B)** Forest plot from multivariable logistic regression analysis with ‘undergoing urinalysis or renal function-related testing within the past 2 years’ as the outcome variable among the CKD high-risk population. **(C)** Forest plot from multivariable logistic regression analysis with ‘attainment of CKD core knowledge’ as the outcome variable in the primary healthcare worker survey data.

In the public survey data, a multivariable logistic regression model was constructed with “having heard of CKD” as the outcome, adjusting for sex, age, education level, and history of chronic disease. Results indicated that having a high school education or above was significantly associated with higher CKD awareness (OR = 2.14, 95% CI: 1.62–2.83, *p* < 0.001), while being aged ≥60 years was significantly associated with lower awareness (OR = 0.68, 95% CI: 0.52–0.88, *p* = 0.004). No significant association was observed between sex and CKD awareness (*p* > 0.05; Figure 4A).

For the CKD high-risk group, a multivariable logistic regression model was constructed with “undergoing urinalysis or renal function testing in the past 2 years” as the outcome. After adjusting for age, sex, education, and disease history, individuals who had previously received health education were more likely to have undergone screening (OR = 1.87, 95% CI: 1.38–2.53, *p* < 0.001), corresponding to a 15% absolute increase in screening probability. Clinically, this implies that approximately 62 additional high-risk individuals per 100 participants would undergo screening if exposed to health education. Similarly, a higher level of CKD core knowledge was associated with a 2.05-fold increase in the odds of screening (95% CI: 1.52–2.77, *p* < 0.001). In this model, the association between high-risk status alone and screening behavior, while attenuated after adjusting for cognitive factors, remained statistically significant (OR = 1.34, 95% CI: 1.01–1.78, *p* = 0.041; Figure 4B).

In the healthcare worker data, a multivariable logistic regression model with “attainment of CKD core knowledge” as the outcome was constructed, adjusting for age, sex, work experience, job category, institution type, and laboratory resource availability. Results showed that healthcare workers with ≥10 years of experience had a significantly higher probability of knowledge attainment compared to those with <10 years (OR = 1.56, 95% CI: 1.18–2.07, *p* = 0.002). Clinical practitioners had higher odds of knowledge attainment compared to public health practitioners (OR = 1.83, 95% CI: 1.39–2.42, *p* < 0.001). Furthermore, working in an institution with adequate laboratory testing facilities was significantly associated with higher knowledge attainment (OR = 1.49, 95% CI: 1.12–1.98, *p* = 0.006; Figure 4C).

To test whether the effect of health education on screening behavior varies by institutional ACR testing capacity, we introduced an interaction term between “receipt of health education” and “availability of ACR testing at the primary care facility.” The interaction was statistically significant (OR = 1.52, 95% CI: 1.12–2.07, *p* = 0.008), indicating that health education is more effective in promoting screening when local testing resources are available. This supports the hypothesis that system capacity moderates the awareness–behavior link (H2).

To facilitate clinical interpretation, we calculated marginal effects (average marginal effects, AME) for key predictors of screening behavior among high-risk individuals based on the logistic regression model. After adjusting for covariates, receiving health education was associated with a 15.2 percentage point increase in the probability of undergoing screening (95% CI: 10.9–19.5, *p* < 0.001). Each one-point increase in CKD core knowledge composite score (range 0–10) increased screening probability by 7.8 percentage points (95% CI: 5.1–10.5, p < 0.001). In contrast, age ≥60 years reduced screening probability by 8.3 percentage points (95% CI: −12.6 to −4.0, p < 0.001). Having a high school education or above increased screening probability by 18.2 percentage points (95% CI: 12.4–24.0, *p* < 0.001). These absolute risk differences provide more intuitive clinically actionable estimates than odds ratios alone.

### 3.5 Distribution of ACR testing and renal biopsy pathological characteristics

A total of 166 valid ACR test results were analyzed. ACR levels showed a markedly right-skewed distribution (Kolmogorov–Smirnov test, *p* < 0.001; Table 3). The median ACR was 6.2 mg/mmol (IQR: 3.2–9.8). Using clinical thresholds, 72 individuals (43%) had ACR < 3 mg/mmol, 66 (40%) had ACR 3–30 mg/mmol, and 28 (17%) had ACR > 30 mg/mmol, with significant differences across strata (Table 3). Stratified by sex, median ACR was higher in females (6.5 mg/mmol, IQR: 3.5–9.0) than males (6.0 mg/mmol, IQR: 3.0–10.0; *p* = 0.04). Albuminuria (ACR ≥ 3 mg/mmol) prevalence was 46% in females and 40% in males (not significant; Table 3).

**Table 3:** Distribution of ACR results and renal biopsy pathological characteristics (*N* = 166).

**Variable**	**Category / Value**	**Statistic / n (%)**	**P value**
ACR testing indicators			
Distribution pattern	Right-skewed	Kolmogorov–Smirnov test	<0.001
Median (IQR), mg/mmol	Overall	6.2 (3.2–9.8)	–
	Female	6.5 (3.5–9.0)	Mann–Whitney *U* = 2,456, *P* = 0.04
	Male	6.0 (3.0–10.0)	
Clinical stratification, *n* (%)	ACR < 3 mg/mmol	72 (43.4%)	–
	ACR 3–30 mg/mmol	66 (39.8%)	–
	ACR > 30 mg/mmol	28 (16.9%)	–
Proteinuria (ACR ≥ 3 mg/mmol), *n* (%)	Female	40/86 (46.5%)	χ^2^ = 1.52, *P* = 0.21
	Male	32/80 (40.0%)	
Renal biopsy pathological spectrum			
Pathological types, *n* (%)	IgA nephropathy	48 (28.9%)	<0.001 (χ^2^ test)
	Minimal change disease	40 (24.1%)	
	Focal segmental glomerulosclerosis (FSGS)	24 (14.5%)	
	Lupus nephritis	22 (13.3%)	
	Membranoproliferative GN (MPGN)	18 (10.8%)	
	Diabetic nephropathy	14 (8.4%)	
Median age by pathology, years (IQR)	IgA nephropathy	49 (45–55)	<0.001 (Kruskal-Wallis test)
	Minimal change disease	42 (39–47)	
	MPGN	60 (58–65)	
Gender tendency by pathology (%)	MPGN (male dominant)	65	–
	Lupus nephritis (female dominant)	70	–

Renal biopsy pathological analysis included the same 166 consecutive cases. Primary glomerular diseases predominated. IgA nephropathy was most common (48 cases, 29%), followed by minimal change disease (40, 24%), focal segmental glomerulosclerosis (24, 14%), lupus nephritis (22, 13%), membranoproliferative glomerulonephritis (18, 11%), and diabetic kidney disease (14, 8%). Proportions differed significantly (Table 3). Age distribution across pathological types also differed significantly (Kruskal-Wallis H = 102.75, *p* < 0.001): median age 49 years (IQR: 45–55) for IgA nephropathy, 42 (39–47) for minimal change disease, and 60 (58–65) for membranous nephropathy. Sex composition varied significantly (Table 3): membranous nephropathy predominantly affected males (65%), and lupus nephritis predominantly females (70%).

### 3.6 Population characteristic patterns based on latent class analysis

Latent class analysis performed on public survey data identified three distinct classes with varying cognitive and behavioral profiles. These results are exploratory, not implying causality. The three-class solution was selected based on lowest AIC, BIC, and aBIC values, and potential misclassification was assessed (mean posterior probability = 0.83). Limitations include lack of external validation and uncertainty in class assignment, which should be considered when interpreting subsequent analyses. The three-class model for the public sample showed optimal fit (AIC = 21483.6, BIC = 21794.2, aBIC = 21601.9) and satisfactory classification accuracy (mean posterior probability = 0.83). Class 1 (30%, “Low Awareness–Low Screening”) had the lowest CKD core knowledge accuracy (30%) and screening rate (19.6%), with disproportionately high percentages of older adults (45% aged ≥60 years) and those with ≤junior high education (40%). Class 2 (45%, “Moderate Awareness–Moderate Screening”) showed moderate knowledge accuracy (50%) and screening rate (33.8%) with mixed demographics. Class 3 (25%, “High Awareness–High Screening”) had the highest knowledge accuracy (80%) and screening rate (80%), and the largest proportion of individuals with ≥high school education (68.1%). Differences in age and education across classes were significant (χ^2^ = 284.7, *p* < 0.001), indicating these are strong determinants of CKD cognition and screening behavior.

Among primary healthcare workers, a three-class solution was also best-fitting (AIC = 17842.1, BIC = 18196.7, aBIC = 17984.3; mean posterior probability = 0.85). Class 1 (20%, “Low Knowledge–Low Management Capacity”) had the lowest knowledge attainment (30%), proactive screening (10%), and regular follow-up (15%), primarily consisting of workers with shorter experience and those in public health roles. Class 2 (50%, “Moderate Knowledge–Moderate Management Capacity”) showed intermediate knowledge attainment (50%), proactive screening (30%), and regular follow-up (40%). Class 3 (30%, “High Knowledge–High Management Capacity”) achieved the highest knowledge attainment (80%), proactive screening (70%), and regular follow-up (75%), characterized by significantly higher proportions of workers with ≥10 years’ experience (60%), clinical positions (80%), and adequate laboratory facilities (70%). Between-class differences were statistically significant (χ^2^ = 319.6, *p* < 0.001), highlighting the roles of work experience, job role, and institutional resources in shaping CKD management capacity (Table 4).

**Table 4:** Latent class analysis identifying population characteristic patterns among public participants and primary healthcare workers.

**Population**	**Latent Class**	**Class Proportion (%)**	**Key Knowledge Indicator**	**Key Behavior Indicator**	**Demographic / Professional Characteristics**	**Model Fit Indices**	**Classification Quality**	**Between-Class Comparison**
Public	3-class model (optimal)	–	–	–	–	AIC = 21483.6	Mean posterior probability = 0.83	–
						BIC = 21794.2		
						aBIC = 21601.9		
	Low awareness – low screening	30%	CKD knowledge accuracy: 30%	Screening rate: 19.6%	Age ≥60 years: 45%	–	–	*χ*^2^ = 284.7, *P* < 0.001
					≤Junior high education: 40%			
	Moderate awareness – moderate screening	45%	CKD knowledge accuracy: 50%	Screening rate: 33.8%	Middle-aged predominant; mixed education levels	–	–	–
	High awareness – high screening	25%	CKD knowledge accuracy: 80%	Screening rate: 80%	≥High school education: 68.1%	–	–	–
Healthcare Workers	3-class model (optimal)	–	–	–	–	AIC = 17842.1	Mean posterior probability = 0.85	–
						BIC = 18196.7		
						aBIC = 17984.3		
	Low knowledge – low management capacity	20%	Knowledge attainment: 30%	Proactive screening: 10%	Shorter working years;	–	–	*χ*^2^ = 319.6, *P* < 0.001
				Regular follow-up: 15%	Public health positions			
	Moderate knowledge – moderate management capacity	50%	Knowledge attainment: 50%	Proactive screening: 30%	Mixed professional background	–	–	–
				Regular follow-up: 40%				
	High knowledge – high management capacity	30%	Knowledge attainment: 80%	Proactive screening: 70%	≥10 working years: 60%	–	–	–
				Regular follow-up: 75%	Clinical posts: 80%			
					Adequate laboratory facilities: 70%			

The three-class solutions for both public participants and healthcare workers were selected based on lowest AIC, BIC, and adjusted BIC values. Mean posterior probability: public 0.83, healthcare workers 0.85, indicating acceptable classification quality. Exploratory analysis only: the identified latent classes describe patterns in cognition and behavior but cannot be interpreted as causal. Differences across classes are descriptive and intended to highlight heterogeneity in awareness, knowledge, and management capacity among the study populations.

### 3.7 Differences in disease detection stage and clinical outcomes across groups with varying cognition and management characteristics

Based on the LCA results, further analysis examined differences in disease detection stage and clinical indicators across the identified public latent classes. Linking latent class membership with ACR outcomes revealed significant differences in the prevalence of albuminuria (ACR ≥ 3 mg/mmol) across classes. While associations are observed, these analyses remain correlational; causality between latent class membership, screening behavior, and disease stage cannot be inferred.

The “Low Awareness-Low Screening” class had the highest prevalence of albuminuria (50%), followed by “Moderate” (35%) and “High” (20%). Among individuals with ACR > 30 mg/mmol, the “Low” class constituted the largest proportion (45%) and the “High” class the smallest (18%), indicating delayed diagnosis in low-awareness groups. Screening behavior incidence showed a significant gradient: 80% in the “High” class vs. 19.6% in the “Low” class, aligning with ACR distribution and suggesting screening as a mediating factor.

Among renal biopsy cases linked to survey data, the “Low” class contributed 45% of cases (exceeding its 30% population proportion), while the “High” class contributed 15% (lower than its 35% population proportion), suggesting delayed diagnosis. At biopsy, the proportion with eGFR <60 mL/min/1.73m^2^ was highest in the “Low” class (50%), intermediate in “Moderate” (30%), and lowest in “High” (10%), especially pronounced in diabetic kidney disease or membranous nephropathy.

From a supply-side perspective, institutions linked to “High Knowledge-High Management Capacity” healthcare workers had a significantly higher proportion of biopsy cases identified during routine examinations or for mild urinary abnormalities (55%) compared to institutions linked to “Low Knowledge-Low Management Capacity” (30%). The latter had a higher proportion of biopsies initiated due to unexplained renal function decline, suggesting that primary care management capacity influences the stage of CKD identification and referral.

The observation that low-awareness class had higher proportion of advanced ACR stages is consistent with H3, where structural constraints (lack of screening) delay diagnosis.

### 3.8 Pilot feasibility outcomes

In the pilot sites, screening uptake increased from 28% (14/50) to 46% (23/50) after 3 months (*p* = 0.04). Referral process completion improved from 42% (21/50) to 65% (33/50) (*p* = 0.01). The workflow integration score rose from 42 to 60 out of 100 (*p* < 0.01). These results support the practical implementability of the model in resource-limited primary care settings.

## 4 Discussion

CKD represents a significant global public health challenge, with its prevalence and associated burden increasing markedly in recent years. An estimated 10% of the global population lives with CKD, with the adult prevalence in China approximating 10.8% (22, 23). The Global Burden of Disease Study 2019 ranked CKD as the 10th leading cause of death and projected it to rise to 5th by 2040 (24). The early stages of CKD are typically asymptomatic, and screening fails to adequately cover all at-risk populations, resulting in most patients being identified only after the disease has progressed to moderate or advanced stages (25). Against this backdrop, this study proposed and evaluated an integrated model for CKD early screening and tiered management, aiming to enhance early detection rates and primary care capacity through systematic risk assessment and testing protocols, thereby helping to curb the rising CKD burden.

Our findings indicate substantial deficits in CKD knowledge and screening behaviors among the public, especially in high-risk groups. Less than one-third of respondents demonstrated sufficient understanding of key CKD concepts, particularly early clinical manifestations and screening methods. These gaps highlight the need for targeted education and behavior-focused interventions. This knowledge gap was especially pronounced among high-risk groups such as those with hypertension or diabetes. These observations align with findings from a 2023 study in Saudi Arabia by Mahmoud et al., which reported approximately 65% of the general public possessed low CKD-related knowledge levels (26). Longitudinal analysis based on the US National Health and Nutrition Examination Survey (NHANES) further showed that despite rising CKD prevalence, awareness among US adults remained stagnant at around 10% between 1999 and 2020, with no substantial improvement (27). Furthermore, studies across diverse countries and healthcare systems consistently indicate that CKD awareness is influenced by various sociodemographic factors—including education, chronic disease history, and health behaviors—exhibiting significant population heterogeneity. For instance, Arnaboldi et al. found statistically significant differences in perceptions of CKD preventability and its risk factors across different social subgroups, underscoring the necessity for stratified, targeted health education interventions (28). In our study, individuals who had undergone renal function assessments (including serum creatinine or albuminuria testing) scored significantly higher on the CKD knowledge assessment. This suggests that health screening behavior may foster a positive feedback loop by enhancing disease risk perception, thereby increasing the likelihood and continuity of future screening participation. An empirical study from Malaysia combining community intervention and media support also demonstrated that a multifaceted health education strategy can effectively improve residents’ CKD knowledge and screening acceptance in the short term, offering a transferable approach for risk identification in low-awareness populations (29). Our study further highlights that stratified knowledge structures and low screening participation rates constitute core barriers to CKD early detection, emphasizing that intervention strategies must concurrently address knowledge dissemination and behavior facilitation. The integrated early screening and tiered management model constructed and validated in this study systematically combines health education, optimized screening pathways, and risk communication. It aims to transcend the limitations of traditional passive screening by establishing a virtuous cycle where improved cognition drives screening participation, which in turn promotes disease identification. For public policy, targeted interventions should prioritize high-risk individuals with low awareness. Concrete recommendations include integrating CKD screening into primary care workflows, establishing ACR testing infrastructure, implementing structured health education programs, and training primary healthcare workers in risk assessment and referral pathways. These measures are particularly relevant for resource-limited regions, ensuring the practical applicability of the integrated model.

The competency of primary healthcare workers in early CKD identification and standardized management directly determines the accessibility and effectiveness of any integrated model in practice. Our study indicates that while targeted training has improved knowledge regarding CKD risk identification and intervention among some primary care doctors, significant competency gaps persist. Survey results show that only 39.6% of primary healthcare workers explicitly recognized the central role of UACR in CKD risk stratification, with an even smaller proportion able to accurately interpret results and guide individualized management, revealing a marked disparity between knowledge and practice. This issue is widely observed elsewhere. A study by Xu et al. in Beijing reported that only 17.2% of high-risk individuals underwent CKD screening, and merely 16.5% of community health service centers possessed UACR testing capability, with most institutions relying solely on eGFR monitoring (30). Limitations in testing modalities not only hinder the timely identification of high-risk CKD individuals but also compromise the precision and continuity of tiered management, thereby affecting the effective utilization of the intervention window. These findings reinforce the argument that capacity building at the primary care level should be a core component of the CKD prevention and control system, with urgent need for structural support specifically in promoting awareness, interpretation skills, and practical application of UACR (31, 32). International experience corroborates that enhancing the responsiveness of primary care in CKD screening cannot rely solely on guideline dissemination. The Italian ALLIANCE project recommends systematically integrating risk education, standardized training, and specialist collaboration mechanisms to effectively standardize UACR testing and referral pathways, thereby narrowing the competence gap between primary and specialty care (33). In constructing our integrated model, we combined screening pathway maps, risk assessment tools, and closed-loop referral processes within a systematic framework to empower primary care practice, achieving some success. However, implementation challenges such as suboptimal referral rates and imperfect follow-up mechanisms persisted, suggesting that individual competency enhancement alone is insufficient to ensure the effective closure of the management chain. Future efforts should explore synergistic strategies based on remote specialist support, task-driven performance incentives, and clinical decision support/alert tools to further improve the sustainable execution and management efficacy of the integrated model at the primary care level.

UACR testing, a key indicator for early glomerular injury detection, is integral to standardized CKD risk stratification and intervention pathways. Our results demonstrate that public screening behaviors substantially influence the actual completion rate of UACR testing. Individuals who proactively participate in health check-ups or express concern for kidney health had significantly higher UACR testing rates than non-participants, indicating a strong link between individual health behaviors and this critical testing step. This finding extends previous research. For example, a large multi-site US study by Stempniewicz (2021) found that while annual eGFR testing reached 89.5% among patients with type 2 diabetes, UACR testing was only 52.9% (34), revealing a persistent systemic gap in early renal injury assessment in clinical practice. Another simulation-based analysis confirmed that increased UACR testing coverage correlates positively with higher CKD identification rates, projecting that raising testing rates from 20 to 100% could increase identification from 6 to 30%. In contrast, our study, based on regional real-world data, systematically reveals for the first time the direct association between individual screening behavior and UACR test completion, providing empirical insight into the behavioral mechanisms affecting testing implementation. This result emphasizes that while optimizing testing technology and screening workflows is necessary for advancing CKD early detection, public cognition and behavioral interventions are equally critical in translating screening intention into actual action (35). Accordingly, our integrated model combines health education, process integration, and primary care capacity building into an operational pathway encompassing population mobilization, test execution, and referral management. Preliminary results suggest potential improvements in UACR testing coverage, though further longitudinal evaluation is required to confirm effectiveness.

Regarding CKD clinical stages and pathological spectra, our study reveals regionally representative distribution patterns. The results indicate a concentration of patients in G1 and G2 stages, with relatively lower proportions in G3 and beyond, presenting an epidemiological picture dominated by insidious early-stage CKD. This trend aligns with findings from a large-scale epidemiological survey in Yunnan Province, which reported G1 and G2 stage proportions of 61.5 and 34.2%, respectively (36). This pattern suggests the presence of a substantial pool of insufficiently identified early cases within our region. Although their renal function indicators may not yet show significant decline, their future risk of disease progression should not be underestimated, highlighting the necessity of strengthening risk-stratified assessment and sustained monitoring mechanisms. Furthermore, integration of renal biopsy data revealed that primary glomerular diseases constituted the predominant pathological type, far exceeding secondary etiologies—a basic characteristic consistent with domestic multicenter study findings (37). This etiological structure indicates a relatively focused CKD pathological spectrum in our region, providing a basis for targeted optimization of early screening strategies and individualized design of intervention pathways. Simultaneously, it points to the current lag in pathological assessment within the actual diagnostic workflow, suggesting the potential utility of introducing more sensitive molecular markers and non-invasive risk prediction tools to achieve a more proactive and precise etiology-oriented management model.

### 4.1 Study limitations

This study has several limitations. First, the cross-sectional design prevents establishing causal relationships between awareness, screening behavior, and clinical outcomes. Second, self-reported data may introduce recall and social desirability bias. Third, the study population is region-specific (Qiandongnan Prefecture), limiting generalizability to other regions. Fourth, while multi-source data were integrated, residual confounding may exist. Finally, the LCA models, although informative, are exploratory and require external validation before broader application. Looking ahead, multicenter prospective studies across broader populations and regions are needed to systematically evaluate key individual- and institutional-type factors influencing CKD cognition, screening, and tiered management outcomes, further validating the generalizability, feasibility, and cost-effectiveness of this model. Particular attention should be paid to the potential roles of emerging technologies—such as digital health tools, remote management platforms, and AI-assisted decision support systems—in enhancing screening efficiency, optimizing clinician-patient collaboration, and enabling precise risk identification; these should be incorporated into future pilot evaluations. Concurrently, sustained systematic training for primary healthcare workers and the public, supplemented by policy incentives and community support, should be pursued to promote the routine and standardized implementation of CKD early screening and tiered management systems. Overall, this study provides a feasible practical framework and data support for regional CKD prevention and control pathways, offering foundational evidence for constructing a locally adapted comprehensive chronic disease management system.

## 5 Conclusion

Focusing on key challenges in CKD early prevention and control in Western China, this study constructed and validated an integrated, informatics-supported intervention model combining public awareness enhancement, primary care screening execution, and clinical management optimization. Integrating survey data from 1,769 public members and 960 primary healthcare workers, together with ACR testing and renal biopsy pathological spectra, the study identified major impediments to early CKD detection and tiered management: insufficient health knowledge dissemination, low screening coverage, significant variations in primary care competency, and uneven testing resource distribution. Results further demonstrate a positive association between screening behavior and cognitive level, and show that ACR testing significantly improves early CKD identification, indicating that intervention strategies should synergistically emphasize behavioral motivation and technical support.

The findings provide preliminary evidence supporting the model’s implementability and intervention value. Model feasibility testing included internal cross-dataset verification and pilot implementation in two township health centers (n = 50 patients), which showed that screening uptake improved from 28 to 46%, referral process completion from 42 to 65%, and workflow integration scores by 18 points on a 100-point index – demonstrating measurable operational feasibility in real-world primary care settings. Concurrently, attention should be directed toward integrated application of digital health tools, multidisciplinary collaboration mechanisms, and primary care empowerment strategies. These efforts should advance the transformation of CKD prevention and control from delayed detection toward proactive identification and precise intervention, ultimately enhancing chronic disease governance capacity at regional and national levels.

## Data Availability

The raw data supporting the conclusions of this article will be made available by the authors, without undue reservation.
